# Perceptions of primary healthcare professionals towards their role in type 2 diabetes mellitus patient education in Brazil

**DOI:** 10.1186/1471-2458-10-583

**Published:** 2010-09-29

**Authors:** Heloisa C Torres, Brani Rozemberg, Marta A Amaral, Regina CA Bodstein

**Affiliations:** 1 Professor, Researcher, PhD- Escola de Enfermagem da Universidade Federal de Minas Gerais-EE/UFMG, Belo Horizonte, Brazil; 2 Professor, Researcher, PhD - Fundação Oswaldo Cruz, Escola Nacional de Saúde Pública, Centro de Pesquisas em Saúde do Trabalhador e Ecologia Humana. Rio de Janeiro, Brazil; 3 Professor, Researcher, PhD - Escola Nacional de Saúde Pública da Fundação Oswaldo Cruz- ENSP/FIOCRUZ. Rio de Janeiro, Brazil

## Abstract

**Background:**

The aim of the current study was to analyze the perceptions, knowledge, and practices of primary healthcare professionals in providing patient education to people with type 2 diabetes mellitus.

**Methods:**

A total of 23 health professionals working in primary healthcare units in Belo Horizonte, Minas Gerais State, Brazil, participated in a focus group in order to discuss their patient education practices and the challenges for effective patient education in diabetes self-management.

**Results:**

The results were categorized as follows: 1) lack of preparation and technical knowledge among the health professionals on some aspects of diabetes mellitus and the health professionals' patient education practices; 2) work conditions and organization; 3) issues related or attributed to the clientele themselves; and 4) diabetes care model.

**Conclusions:**

This study highlights the importance of reorienting the patient education practices, health professionals' skills and work goals, and evaluation of the educational interventions, in order to establish strategies for health promotion and prevention and control of the disease.

**Descriptors:**

Health Education; Prevention of Diabetes Mellitus; Primary Healthcare

## Background

The incidence and prevalence of type 2 diabetes mellitus are increasing in epidemic proportions and impacting the 30 to 69-year age bracket [1]. The increasing morbidity and mortality in this population group and the complexity of diabetes treatment, including dietary restrictions, use of medication, and associated chronic complications (retinopathy, nephropathy, neuropathy, cardiopathy, neuropathic foot, and others) emphasize the need for effective educational programs and training for health professionals, in order for them to meet this demand adequately. This is one of the current challenges for public health, especially in primary care. Patient education programs and strategies can contribute to behaviour change and improve metabolic control and self-monitoring of skills for the individual with diabetes mellitus to make treatment decisions [2,3]. Education for people with diabetes is a therapeutic approach that motivates individuals to acquire knowledge and develop skills that facilitate self-management of care [4,5].

Several authors [6-9] have added that to achieve effective education for people with diabetes requires providing health professionals with training, current knowledge on the disease, pedagogical skills, effective communication, listening, and understanding, as well as the capacity to negotiate with individual patients and use dynamic and interactive strategies to reduce the barriers to high-quality individual care.

Current efforts to improve the quality of diabetes care are based on knowledge of factors associated with the health professionals, including the limitation of their knowledge and skills concerning the disease, organization of their educational practices, and quality of provider-patient interaction based on cultural, social, and cognitive understanding and language. Several studies[8-10] have suggested that such factors act as barriers or facilitators for implementing patient education for self-care and that they affect the choice of individual patients' treatment choices. However, relatively few studies have been performed to identify these factors.

The study thus focused on a group of health professionals working in primary care and academia and involved in health service practices. When designing the patient education process in diabetes, they identified the need to investigate the existing educational practices in the health service and the results for individual patients. This realization raised the demand for reorganizing the patient education program in diabetes, analyzing each health professional's skills, the work goals in patient education activities, and the results of interventions in order to establish strategies for health promotion and prevention and control of the disease.

The current study thus proposes to analyze perceptions, knowledge, and practices by primary healthcare professionals' in providing patient education to people with type 2 diabetes mellitus.

## Method

The research was designed as a case study with a descriptive/exploratory qualitative approach. The study was developed and conducted in two primary care units on the East Side of Belo Horizonte, Minas Gerais State, Brazil, from April to June 2009. The criterion for selecting the recruitment sites was ease of access due to their link to university services. The health professionals were included in the study because they: were working with individual and group patient education for people with diabetes, had more than two years of experience with educational practices, and showed experience and interest in diabetes education. The sample thus consisted of 23 health professionals with university training that work in primary care, particularly involved in diabetes care, and that aim to implement a model educational program for people with diabetes.

The data were collected using the focus group technique and filling out an identification form for health professionals using specific instruments. The focus group meetings included 10 to 12 participants, i.e., a kind of interview or conversation in homogeneous groups, designed to obtain information on a specific theme [11,12]. The focus group explored barriers and facilitators for successful patient education in diabetes. The identification form provided a description of the participants, including: gender, age, schooling, profession, time on the job, and experience with individual and group patient education practices in diabetes.

The participating health professionals worked in the fields of nutrition, physical therapy, medicine, and nursing. Their time on the job ranged from 9 to 28 years, and they were all women with experience working with group and individual patient education for people with diabetes.

Two focus group meetings were held in each primary care unit, lasting one hour each and involving an average of 10 to 12 health professionals, totalling four meetings.

The group interviews followed a focus group script with the following themes: educational practices (facilitating factors and barriers), integration between patients and the health team, essential elements for developing and continuing patient education, and proposals for improving the patient education programs. Audio recordings were made of the group discussions.

To ensure the participants' anonymity, the interviews were numbered 1, 2, 3, 4,....10.

The material was recorded, systematized, and categorized to build a database, considering recurrent and frequently expressed opinions, disagreements, and consensuses. The data were then processed and interpreted according to the thematic analysis focus, adapted by Minayo [13].

The principal category emerging from analysis of the material relates to difficulties and barriers experienced by the professionals in performing more effective work. Within this category we created subcategories in which we grouped the answers related to: 1) work conditions; 2) work organization; 3) lack of the professionals' technical preparedness in relation to diabetes; 4) issues related to individual patient conditions; and 5) issues related to the patient education practice itself. This last set of factors was the central object of our analyses, including discussion of: a) the professionals' involvement in the patient education practice; b) limited provider-patient integration; c) reduction of the discussion on diabetes to test results and prescription changes; and d) issues pertaining to participatory methods.

The study was approved by the Institutional Review Board of the Federal University in Minas Gerais (UFMG) and the Belo Horizonte Municipal Health Secretariat, having complied with all the requirements of Brazilian National Health Council Ruling 196/96, which deals with ethical issues in research involving human beings.

## Results

The findings were grouped according to aspects highlighted by primary healthcare professionals. In the discussions, all the professionals reported the *"difficulties and barriers" *in dealing with daily situations related to group education strategies for more effective work with diabetes.

Our analysis showed that from the health professionals' point of view, the principal barrier to the effectiveness of diabetes care was the professionals' inadequate knowledge on how to manage diabetes and group education, as reflected in the following quotes:

### Lack of knowledge on diabetes among the health professionals

The professionals reported that they felt insufficiently prepared to conduct educational practices for people with diabetes, pointing to gaps in their knowledge on the disease, the importance of diabetes management, and problems in pedagogical methodologies as aspects that hinder more effective results in patient education for diabetes self-management.

**E1**: "*I feel poorly prepared in relation to the [patient] education dynamics and teaching techniques [...] I don't feel trained for health education or developing knowledge on the disease"*

Another aspect frequently identified in the discussion relates to the health professionals' perception of the work conditions in which primary care is provided.

### Perception of Work Conditions

Low wages, limited physical space for patient education activities, and limited access to the health centre's coverage area were perceived by the health professionals as factors that affected the patient education practice in diabetes. They reported the need to establish partnerships to use places other than the health centre such as churches, homes for the elderly, and neighbourhood associations in order to ensure comfortable areas with the necessary privacy for the patient education activities.

**E3**: *"The physical space is very limited [...] there's no way to conduct the educational activities [...] the coverage area is huge, and for some patients it's hard to reach the health centre and participate in the groups."*

In the discussions, the participants remarked on the scarcity of educational materials, lack of structure in the educational process, and lack of recognition of the groups' importance in patient education for self-care.

### Perceptions of the work organization

Some professionals pointed to the absence of a clear proposal in the diabetes care service and remarked on the lack of time, the work overload, and lack of planning in the patient group activities.

**E4: ***"The groups are large, involving talks with too many people. It's very difficult to orient so many people all together [...] with meetings once a week [...] the group becomes tiresome, repetitive*, *unproductive*, *uninteresting, always the same people [...] the group doesn't have a start or finish."*

Thus, the problems detected by the majority of the health professionals refer to the services' organizational structure: lack of time and availability to participate in the groups due to the large number of individual patient consultations and the lack of training and motivation for professionals to lead groups.

The health professionals' motivation was considered an important factor, according to quotes from the participants, and appeared more frequently among physicians and nurses that participated in patient education activities. Motivation was viewed as a catch-all term for issues related to professional interests, intentions, and awareness.

The participants identified several economic, cultural, and social patient-related factors that impact the continuity of patient education activities in diabetes.

### Conditions of individuals with type 2 diabetes

The discussions revealed the difficulties in adhering to patient education for self-care, and some health professionals confirmed that economic, cultural, and social factors affected individual attitudes in diabetes self-management. The barriers identified by health professionals among individual patients included lack of time to adhere to healthy life habits, lack of money, absence of appropriate places for physical exercise, and individual passivity towards treatment.

Problems in provider-patient communication included health professionals' lack of understanding of the social context, inattention to specific knowledge, language differences, and insufficient consultation time for patients to be heard and express their doubts, knowledge, and difficulties in understanding medical terminology. According to this interpretation, information alone is not effective for patients to decide and take an active part in the treatment. Meanwhile, health professionals' role in routine patient education in diabetes is predominantly paternalistic.

**E6: "***Sometimes the language issue, attitude [...] and the inability to construct knowledge together with the [patient] population [mean that] some professionals feel unprepared for [patient] education [in diabetes] and lack the skills to recognize their own limitations."*

The health professionals reported that it is important to improve individual patient education by working on cultural and social issues and developing reflexive listening to foster education in self-care and help patients realize that their own actions can make the difference in diabetes treatment.

### Patient education practice in diabetes

In this category, participants emphasized the limited involvement by the health professionals in patient education, resulting in repetitive and mechanical work and the lack of an interdisciplinary approach. The professionals felt the need to interact more with individual patients and participate in the groups with other colleagues, jointly discussing what can be done to improve daily patient education practice. Some admitted that they were not concerned about how they should lead the groups and educational activities.

**E8**: *[...] we don't educate the clientele very well, and this limited education doesn't help with diabetes prevention and control [...] it's important for the health professional to become active and involved in the educational practice [...] to be available and take interest in teamwork."*

**E2**: *"[...] I've tried to improve the patients' blood glucose levels [...] but we haven't achieved the objectives of educational work [...] The professionals' effort is very limited, and they need to share [the work] with other professionals."*

The health professionals commented on the importance of receiving training in patient education for self-care, defining their skills and tasks within the team in order achieve decentralization from the physician/nurse figure in diabetes care and seek an interdisciplinary approach for developing a model educational program.

According to the participants' discourse, the second greatest difficulty in education was the limited integration between the health professionals and patients, which should be based on cultural, social, cognitive, and linguistic comprehension.

**E10**: *"The groups currently lack involvement by all the professionals. Few lead the group [activities with patients], while individual patients demand participation by other professionals [...] and this situation hinders the educational practice."*

Additionally, the health professionals interviewed here were not concerned about the cognitive, motivational, and emotional barriers in the provider-patient relationship that interfere in self-care and self-management of the illness.

The participants highlighted the importance of involving the team in the educational practice; they emphasized that individual health workers should know their competency and the limits of their scope of work. According to the health professionals, the group debates focus only on test results and changing prescriptions, with poor use of the space for dialogue, which becomes "*tiresome and boring*", a frequently reported difficulty in patient education in diabetes, as illustrated below.

**E7**: "*the group focuses mainly on changing prescriptions, obtaining the medication, and having the physician check the test results [...] and discuss the difficulty in understanding the prescription, which is sometimes long and drawn out, [...] all of which becomes tiresome and boring for the health professionals."*

The participants also recognize that the health professionals and patients should collaborate to develop participatory strategies in order to improve the odds of managing the disease and reduce the physical, psychological, social, and economic consequences of diabetes, as described next.

### Change in the Diabetes Care Model

In the educational process, it would be appropriate for health professionals to include planning activities that promote group work in shared knowledge-building. The professionals interviewed here acknowledged the need to improve diabetes care, as described in the following interview:

**E8: "***Group discussions with patients should be based on the population's needs with the objective of obtaining more and interested spontaneous individual participation. Thus, the educational process becomes more effective if it is based on the community's wishes and needs."*

**E9: **"*When working in groups involving professionals with different knowledge sets, you can't bring a pre-formatted approach for the individual patients. It has to be something that the population suggests, based on their needs, to exchange knowledge. The team's involvement is important; professionals need to be aware of the competencies and limits of their co-workers from other professions."*

The health professionals also mentioned the importance of assessing the patients' economic situation in order to orient their eating plan and reinforce the provider-patient contact, communication between the various persons involved in the process, the language used by the health professional, and the space for discussing and elaborating didactic materials to be used in patient education. They highlighted that patients should be well informed about their illness in order to achieve effective treatment results and know how to manage their care and obtain control of the treatment.

## Discussion

The current patient education process is based on health professionals "transferring" information on diabetes (the disease), rather than a more comprehensive and effective educational approach. The approach should focus on the need to establish a dialogue and thus on the capacity to hear the needs and demands of the groups with whom one is working. The target of the process should also be knowledge on living and work conditions and life habits and lifestyles, which would tend to foster patient education focused on self-management, i.e., training individuals for self-control over the determinants of their health [6,11].

The health professionals' point of view relates to activities that have been implemented within inadequate structures (hasty consultations with symbolic configurations and specific power relations) through individual or group initiatives that have produced little progress. They emphasized that the lack of time, problems with the professionals' motivation, lack of financial incentives and didactic materials, and individual patient passiveness towards treatment limit the effectiveness of patient education and the implementation of an educational program in primary care and daily patient education [5,10].

The health professionals' discourse suggests that they are blaming the victims, i.e., incriminating the patients for their own disease. The professionals thus tend to reproduce a restricted and normative view and practice in relation to the health/disease process. Meanwhile, their discourse indicates discomfort towards such practices and a perception of their own limited effectiveness.

The study's results indicate that the health professionals are aware and want to modify their behaviour in patient education and complain of a lack of training for such change, specifically in relation to patient education in diabetes. Some authors[2,14] have emphasized that any intervention to improve health services should train the health team in a constant effort to improve the social relations appearing in the services' daily activity, from a critical and reflexive perspective towards the work process. Thus, in addition to the basic investment in health work conditions and organization, it is clearly necessary to invest in training health professionals in primary care and education, in close collaboration with researchers, leading to innovative products for the services and relevant new challenges for the academic community.

The findings in the literature [5,15] indicate that the level of knowledge required by people with diabetes is associated with health professionals' ability to listen and their own recall capacity, managed according to formal principles such as "the doctor asks and the patient replies". Patients are rarely in a position to build knowledge by asking about their treatment or their own experiences, comparing, analyzing, or verbalizing their own day-to-day knowledge about health education.

Additionally, several authors [16-18] state that people with diabetes report satisfaction in the interaction of forms of knowledge built through exchange of experiences and knowledge between the health team and themselves. Appreciation of the social issues and setting is crucial for diabetes treatment and patient education [14-17]. It is thus necessary to reinforce a perspective that discusses the social determinants of health. Consistently, it is necessary to expand knowledge and practices to deal with these social determinants in primary care services, through more reflexive health promotion strategies and programs and patient education practices.

The challenge is to expand an attitude of listening to and considering the relevance of patients' experiences and perceptions and asking them what they need in order to improve their self-care. The task of health professionals during patient education for self-care involves evaluating and understanding individual patients, providing them with emotional and clinical support and knowledge and skills to achieve the treatment objectives, helping them discover and develop autonomy to deal better with their illness [14,15].

This study focused exclusively on health professionals with the aim of implementing a patient education model for diabetes within the primary care setting. The findings are consistent with the literature in relation to difficulties in patient education in diabetes [18-20].

In summary, health professionals need to reflect on educational practices and how they are constructed in the field of education and health. In the health service analyzed here, current patient education strategies in diabetes are not favouring self-care and patients' control of the disease, and there is a lack of progress in these activities. Health professionals should seek to develop health education skills and admit the difficulties, limitations, and slow and gradual nature of the learning process in order to obtain the "results" that allow improving patient education in diabetes.

The suggested improvements for achieving a systematized patient education process include updating the health professionals' knowledge, combining aspects from education and health promotion in order to include professionals that are capable of reflecting on and intervening in their educational actions, with individual patients as their partners in reorienting the educational practice. Such health work organization aims to assign responsibility to primary care managers, who should seek to induce, plan, and encourage the adoption of more comprehensive educational strategies and practices, provide better training for the health team, work with an evaluative perspective toward their practices to make education an important therapeutic tool in health professionals' practice, and help them improve the work methodology and information for patient self-care in type 2 diabetes mellitus [2].

## Conclusions

Participation in the focus group provided the health professionals with the opportunity to reflect on the patient education process and the difficulties in establishing and maintaining the practice due to their lack of specific training in health education, a gap that has persisted from their original undergraduate training to the present in their work as professionals. This gap appears in the patient education program, where the absence of organization and planning in the educational practice jeopardizes the quality of individual patient care.

We observed that the focus group results allowed health professionals to assume their role as educational protagonists, producing emancipative knowledge, encouraging reflection and the capacity for critical analysis, including patient education in diabetes as one of the determinants of successful self-care by patients, in the attempt to provide individual patients with therapeutic education for self-management of the illness.

## Competing interests

The authors declare that they have no competing interests.

## Authors' contributions

HCT and RCB initiated this study and conducted the literature review and key informant interviews and tabulated the key findings. HCT and BR and MA drafted most of the paper. All of the authors read and approved the final manuscript.

## Pre-publication history

The pre-publication history for this paper can be accessed here:

http://www.biomedcentral.com/1471-2458/10/583/prepub
